# Climate change effects on plankton recruitment from coastal sediments

**DOI:** 10.1093/plankt/fbad060

**Published:** 2024-02-15

**Authors:** Per Hedberg, Markus Olsson, Helena Höglander, Volker Brüchert, Monika Winder

**Affiliations:** Department of Ecology, Environment and Plant Sciences, Stockholm University, Stockholm, Sweden; University of Helsinki, Tvärminne Zoological Station, 10900 Hanko, Finland; Department of Ecology, Environment and Plant Sciences, Stockholm University, Stockholm, Sweden; Department of Ecology, Environment and Plant Sciences, Stockholm University, Stockholm, Sweden; Department of Geological Sciences, Stockholm University, Sweden; Bolin Centre for Climate Research, Stockholm University, Stockholm, Sweden; Department of Ecology, Environment and Plant Sciences, Stockholm University, Stockholm, Sweden; Bolin Centre for Climate Research, Stockholm University, Stockholm, Sweden

**Keywords:** resting stage, recruitment, emergence, phytoplankton, zooplankton, dinoflagellate, cyanobacteria, diatoms, copepods

## Abstract

In highly seasonal systems, the emergence of planktonic resting stages from the sediment is a key driver for bloom timing and plankton community composition. The termination of the resting phase is often linked to environmental cues, but the extent to which recruitment of resting stages is affected by climate change remains largely unknown for coastal environments. Here we investigate phyto- and zooplankton recruitment from oxic sediments in the Baltic Sea in a controlled experiment under proposed temperature and light increase during the spring and summer. We find that emergence of resting stage differs between seasons and the abiotic environment. Phytoplankton recruitment from resting stages were high in spring with significantly higher emergence rates at increased temperature and light levels for dinoflagellate and cyanobacteria than for diatoms, which had highest emergence under cold and dark conditions. In comparison, hatching of copepod nauplii was not affected by increased temperature and light levels. These results show that activation of plankton resting stages are affected to different degrees by increasing temperature and light levels, indicating that climate change affects plankton dynamics through processes related to resting stage termination with potential consequences for bloom timing, community composition and trophic mismatch.

## INTRODUCTION

The seasonal timing of life-history events, such as emergence, growth and reproduction is largely determining population dynamics with consequences for community composition and trophic coupling (Sommer *et al*., 2012). The identification of factors and underlying mechanisms that determine life-history events is important for predicting trajectories of individual populations under climate change (Ji *et al*., 2010). Timing is of utmost importance for planktonic organisms in seasonal environments to match favorable environmental conditions and resource availability (Edwards and Richardson, 2004; Winder and Schindler, 2004; Cloern and Dufford, 2005). While most of the climate research on plankton bloom dynamics focuses on the pelagic life stage, a large majority of plankton species overwinter as resting stage in the sediment (Marcus and Marcus, 1998; Cottingham *et al*., 2021). The resting life stage is, however, often overlooked in climate change research, although emergence of planktonic organisms originating from resting stages from the sediment is a key driver for pelagic population dynamic and community composition (Cáceres, 1998; McQuoid and Godhe, 2004; Jerney *et al*., 2022). Therefore, interactions of resting stage recruitment with changing climate conditions should be considered as a driver for bloom formation.

Some plankton species produce resting stages or cysts that when environmental conditions deteriorate (Hansson, 1996; Holm *et al*., 2018) usually sink to the bottom where they remain dormant in the sediment (Ellegaard and Ribeiro, 2018; Holm *et al*., 2018). Vegetative cells originating from resting stages and hatchlings from resting eggs, may emerge prior to the next productive season but they can also remain viable in the sediment for several years, functioning as an insurance for long-term species and genetic diversity (De Meester, 1993; Cáceres and Tessier, 2003; Sanyal *et al*., 2022). The termination of the resting phase is often linked to environmental cues of seasonal changing temperature and light conditions (Gyllström and Hansson, 2004; Ellegaard and Ribeiro, 2018). Photoperiod is a key factor to induce germination in resting stages of a wide variety of phytoplankton, like cyanobacteria, dinoflagellates, green algae and diatoms (Agrawal, 2009), with already low light intensity inducing germination of resting stages. In addition, temperature is a key driver for initiation of emergence from resting stages in both phyto- and zooplankton species, with variable and species-dependent temperature optima for emergence from dormancy (Berasategui *et al*., 2013; Jones and Gilbert, 2016; Ellegaard and Ribeiro, 2018). Climate change and loss of ice cover are altering the seasonality of temperature and light cues that trigger emergence of resting stages with consequences for trophic coupling (Winder and Schindler, 2004; Thackeray *et al*., 2016; Hjerne *et al*., 2019). However, the response to changing environmental conditions may be variable among plankton taxa, depending on the type of resting stage and factors activating growth (Adrian *et al*., 2006).

Understanding underlying processes of life stage transition and particularly emergence from resting stages to vegetative or active pelagic stages and their contribution to bloom inoculation is important for predicting plankton phenology (Cottingham *et al*., 2021). Plankton blooms are either initiated from pelagic life stages in the water column or from benthic resting stages (McQuoid and Godhe, 2004; Holm *et al*., 2018). The contribution from resting stages to the open water column can significantly affect plankton bloom dynamics (Stahl-Delbanco, 2003). Earlier emergence is beneficial if it allows for reduced competition for resources, extension of the growing season, or redistribution of cells to the upper water column during seasonal mixing (Cottingham *et al*., 2021). Emergence of plankton from the sediment may also be unrelated to climate induced change in abiotic conditions and strongly related to the type of resting stage (Lennon and Jones, 2011). An improved understanding of how climate change will affect the transition from the inactive benthic stage to the active pelagic stage will allow us to better predict drivers of plankton bloom dynamics.

Here we investigate climate-induced factors, such as changes in temperature and light levels on the emergence of phyto- and zooplankton from a coastal oxygenated sediment during spring and summer. We use sediment from the Baltic Sea, a highly seasonal system with distinct spring and summer plankton blooms. The information obtained in the present study allows us to identify the magnitude and timing of benthic release of planktonic organisms and to better predict plankton bloom dynamics. Moreover, the results contribute to an improved quantification of how climate change is altering biologically mediated carbon coupling between sediment and water column habitats and implications for trophic coupling.

## MATERIAL AND METHODS

Twenty-four sediment cores were collected with a Multicorer (K.U.M. Umwelt und Meerestechnik, Kiel GmbH), fitted with 4 plexiglass tubes measuring 10 cm (inner diameter) and subsampled with plexiglass tubes (25 cm high, 7.4 cm inner diameter with a surface area of 0.004 m^2^) from a depth of 21 m in the northern Baltic Sea proper (58°50´N, 17°33´E). These smaller tubes were used for the experiments and hereafter called microcosms. The ratio of sediment to overlaying bottom water (451 ml) in each subsample was about 1:1. The microcosms were kept cold and dark from collection until being placed in a temperature-controlled room set to *in situ* bottom temperature for 24 h to allow any suspended material to settle to the bottom. Before deployment of the multicorer, bottom water was collected with a Niskin bottle, and temperature, oxygen and light intensity was measured at 20 m depth by use of a CTD (Sea & Sun Technology GmbH). Sampling was conducted once in the late June 2019 (summer) and early March 2020 (spring). Light and oxygen measurements at 20 m were 2.73 μE m^−2^ and 7.26 ml l^−1^ in spring, and 1.69 μE and 8.83 ml l^−1^ in summer, respectively. In comparison, light values below the water surface were 471 μE m^−2^ in spring and 1042.46 μE m^−2^ in summer, respectively.

Before the experimental start, water overlaying the sediment was siphoned off with syringes as close as possible to the sediment, leaving approximately 5 mm of water. Siphoning off the initial water did, however, not completely remove adult *Eurytemora* individuals in summer (Fig. S1). Thus, the experiment does not allow for separation of *Eurytemora* nauplii recruitment from the sediment or hatching from subitaneous eggs and the *Eurytemora* data in summer are excluded from the analysis. Sterile, filtered (0.2 μm) bottom water from the collection site was drip-fed onto polystyrene foam disc, placed in each microcosm, to avoid resuspension of the sediment. After re-filling the microcosms with water, they were covered with parafilm with small holes in order to minimize evaporation but also to allow for gas exchange and light aeration through a single thin nylon tube per microcosm. Care was taken to allow minimal aeration of the upper few centimeters of the water to avoid any resuspension of the sediment. The 24 microcosms were placed in incubators divided into four abiotic factor treatments, with six replicates each: control CTRL was kept dark and at *in situ* bottom temperature (3 °C in spring and 9 °C in summer), elevated temperature (T), kept dark and subjected to an increase of +2 °C, light (L), subjected to the same temperature as CTRL and to weak green light emitting 0.5 μmol m^−2^ s^−1^ (Nascimento *et al*., 2008), and a combination of elevated temperature of +2 °C and weak green light (T+L). Treatment T was covered with a thick, dark plastic sheet to block all possible light from entering. A +2 °C temperature increase was based on average projected temperature increase for the Baltic Sea by the end of this century and weak green light representing underwater light change due to loss of sea ice (HELCOM, 2021).

Incubation of the microcosms lasted for two weeks and samples were taken at day 7 and day 14 of the experiment. Sampling was done by extracting the overlaying water in each microcosm with syringes, then fixed with acid Lugol’s solution and placed in clear flasks covered with parafilm to prevent evaporation. Samples were allowed to settle for 6 days, then reduced to < 100 ml by carefully extracting the upper part of the sample and transferring the remaining part to clear 100 ml PVC bottles for storage in darkness at 4 °C. At the end of the experiment, sediment from all microcosms was sieved through a 500 μm mesh for identification of benthic macrofauna to determine potential impacts through bioturbation or predation, which did not differ between treatments. All macrofauna retrieved was living, suggesting that the sediment remained well oxygenated throughout the experimental duration.

Identification and quantification of phytoplankton cells followed the Utermöhl method (Edler and Elbrächter, 2010) with a sedimentation chamber of 100 ml. All samples were allowed to settle for 48 h according to standard guidance (HELCOM, 2017). Phytoplankton were identified to either class (dinoflagellates < 20 μm), order (dinoflagellates > 30 μm), genus (diatoms and cyanobacteria) or dinoflagellate cysts, using http://nordicmicroalgae.org/galleries/HELCOM-PEG (2021). Carbon content are based on Olenina *et al*. (Olenina *et al*., 2006) for size classes 27 – 40 and 20 – 27 μm. Zooplankton were identified to genus level using Telesh *et al*. (2015). Nauplii biomass was calculated on naupliar stages I-III using established biomass factors of 2 μg wet weight individual^−1^ for *Acartia* and *Eurytemora*, and 3 μg wet weight individual^−1^ for *Temora* (SMHI, 2023). Phytoplankton germination and zooplankton emergence, and carbon content were calculated as cell and nauplii per m^2^, respectively.

To investigate differences in composition of the phyto- and zooplankton community, we used the generalized linear model (GLM) framework in the mvabund package of R 2.14 (R Core Team, 2022) and *manyglm* function with negative-bimodal distribution (Wang *et al*., 2012). The model fits a separate GLM to each taxon with the specified predictor variables simultaneously in a multivariate framework. Model assumptions were checked visually by plotting the residuals vs fitted data. Goodness-of-fit was performed using analysis of deviance (Dev) with likelihood ratio tests (LRT) to test differences between weeks, seasons and treatments on the phyto- and zooplankton community data using the *anova* function on the *manyglm* model output. To test which taxa contributed to significant difference, we used *anova* with ‘adjusted’ univariate P-values, which performs univariate tests for each taxon separately from the multivariate model output. To identify differences in number of emergence between treatments for individual taxa we used negative-binomial regression (*glm.nb*) in the MASS package. Goodness-of-fit was tested with a chi-square test, followed by simultaneous pairwise comparisons using Tukey’s HSD test.

## RESULTS

**Fig. 1:**
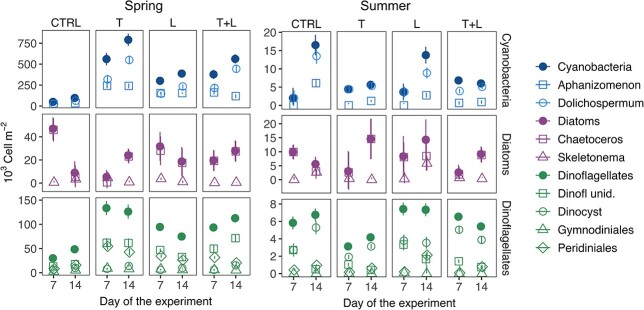
Mean (± S.E.) of phytoplankton cell germination from Baltic Sea coastal sediments at day 7 and 14 of the experiment during the spring (March) and summer (June) for cyanobacteria, diatoms and dinoflagellates. Cell counts are shown for the following treatments: control (CTRL) kept dark at *in situ* temperature (3 °C in spring, 9 °C in summer), an increase of +2 °C (T), weak green light (L), and a combination of elevated temperature and light (T+L). Filled symbols are total counts of the respective taxonomic groups, open symbols are the dominating taxa, contributing to more than 95% of total counts.

Phytoplankton emergence was dominated by cyanobacteria (*Aphanizomenon*, *Dolichospermum*), dinoflagellates (*Gymnodiniales*, unidentified dinoflagellates, *Peridiniales*), and diatoms (*Chaetoceros, Skeletonema*). We observed only few cells of the cyanobacteria *Nodularia* in summer and of the diatom *Coscinodiscus* and *Thalassiosira* in spring and excluded these from the downstream analysis. For phytoplankton, the treatment effect was similar for week 1 and 2 in both seasons (GLM, Dev > 1.49, p > 0.4), although cell counts varied for some taxa (Fig. 1). Overall, emergence of phytoplankton cells was highest in the spring for cyanobacteria followed by dinoflagellates and diatoms, while in summer only few phytoplankton cells emerged.

**Fig. 2:**
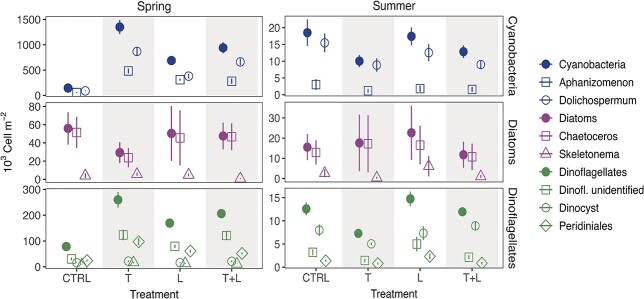
Cumulative abundance of phytoplankton cells (mean ± S.E.) from Baltic Sea coastal sediments during the spring (March, left column) and summer (June, right column) period for cyanobacteria, diatoms and dinoflagellates. See Fig. 1 for treatment description. Filled symbols are total counts of the respective taxonomic groups, open symbols are the dominating taxa, contributing to more than 95% of total counts. Note different y-axis scales for spring and summer.

In spring, cumulative cell counts of total cyanobacteria over the two weeks period increased significantly in all treatments compared to the CTRL (Tukey’s HSD, Z > 12.02, p < 0.001) and reached the highest cell abundances in T (1350.6 vs CTRL 145.6 × 10^3^ cells m^−2^) (Fig. 2). Total cumulative cyanobacteria cell counts were also significantly higher in T compared to the L and T+L treatments (Z > 2.81, p < 0.026). Cumulative cell counts of *Aphanizomenon* were significantly higher at T, L and T+L treatments compared to the CTRL (Tukey’s HSD, Z >8.56, p < 0.001), with averages increasing from 56.5 × 10^3^ cells m^−2^ (CTRL) up to 482.1 × 10^3^ cells m^−2^ at T (Fig. 2). Similarly, *Dolichospermum* cell emergence was low in the CTRL (89.1 × 10^3^ cells m^−2^), higher in the L treatment (380 × 10^3^ cells m^−2 1^, Z = 9.90, p < 0.001) and almost ten times higher in the T and T+L treatments with cumulative counts over the two-week period of 868.5 × 10^3^ cells m^−2^ at T (Z > 13.69, p < 0.001). The number of cumulative cells of total diatoms and the diatom species *Chaetoceros* and *Skeletonema* was not affected by any treatment (Z < 0.78, p > 0.81). *Chaetoceros* dominated the cell counts with average cumulative spring abundances over all treatments of 41.8 × 10^3^ cells m^−2^ compared to 3.9 × 10^3^ cells m^−2^ for *Skeletonema*.

Similar to cyanobacteria, total dinoflagellates increased significantly in all treatments in spring and reached the highest cell abundances in the T and T+L treatments (Z > 6.05, p < 0.001; 259.6 × 10^3^ cells m^−2^ at T vs CTRL 78.6 × 10^3^ cells m^−2^). *Peridiniales* and unidentified dinoflagellates dominated, reaching three- to fourfold higher abundance in the T, L and T+L treatment, up to 97.2 and 123.4 × 10^3^ cells m^−2^ at T, respectively, compared to the CTRL treatment (Z > 4.6, p < 0.001). *Gymnodiniales* and cysts of dinoflagellates had low abundances (< 22.1 × 10^3^ cells m^−2^), although *Gymnodiniale*s were significantly higher in the T treatment compared to the CTRL (Z = 4.47, p < 0.001). In summer, total cyanobacteria and dinoflagellate cell counts were significantly lower in the T compared to the CTRL treatment (Z = -2.72, p = 0.007, Z = -4.20, p < 0.001), while no significant differences between the treatments and CTRL were found in diatoms and respective taxa (Fig. 2).

**Fig. 3:**
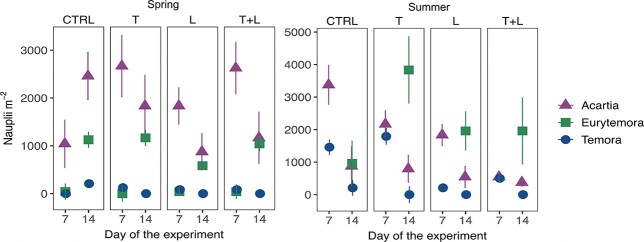
Mean (± S.E.) abundance of zooplankton nauplii hatchlings from Baltic Sea coastal sediments at day 7 and 14 of the experiment during the spring (March) and summer (June). See Fig. 1 for treatment description.

**Fig. 4:**
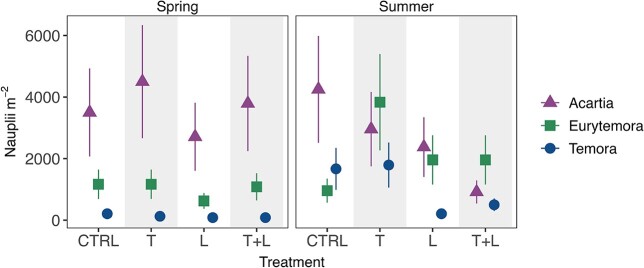
Cumulative abundance of copepod nauplii (mean ± S.E.) from Baltic Sea coastal sediments during the spring (March, left column) and summer (June, right column) period. See Fig. 1 for treatment description.

Zooplankton emergence was dominated by copepod nauplii, while only few individuals of cladoceran and rotifer taxa were detected (n = 8; data not shown). Nauplii were dominated by *Acartia* (59% of total nauplii counts), *Eurytemora* (30%), and *Temora* (11%). In addition, few individuals of *Centropages*, *Cyclops* and *Pseudocalanus* were detected, which are not considered further in the downstream analysis. Treatment significance is not estimated for *Eurytemor*a nauplii in summer because of the presence of adult individuals (see method section). In spring, *Eurytemora* nauplii emergence was significantly higher in week 2 (Dev = 14.03, p = 0.003) and lower for *Acartia* and *Temora* nauplii in week 2 in summer (Dev > 4.45, p < 0.03) (Fig. 3). Cumulative nauplii abundance over the two weeks was about five times higher in summer for *Temora* compared to spring (Dev = 4.5, p = 0.013), increasing from average 125 to 500 nauplii per m^−2^), but did not vary between seasons for *Acartia* (Dev = 2.09, p = 0.09), reaching mean values of 3875 nauplii per m^−2^ in CTRL (Fig. 4). Cumulative nauplii abundances for copepod genera were similar across treatments in spring and summer (Dev < 1.04, p > 0.3), except in summer *Acartia* nauplii were higher in CTRL (4250 ind. m^−2^) than T+L (916.7 nauplii per m^−2^, GLM, p = 0.004), and *Temora* nauplii were higher in the CTRL and T than L (mean CTRL and T 1729.2 vs L 209.3 nauplii per m^−2^, GLM, p < 0.017).

Cumulative emergence of total phytoplankton carbon from the sediment in spring was more than threefold for the T treatment and more than twofold at L and T+L treatments, ranging between 95.5 and 152.6 μg m^−2^ compared to the CTRL emergence of 39.0 μg m^−2^ (Z > 6.68, p < 0.001) (Fig. S2). This pattern was driven by increased emergence of cyanobacteria and dinoflagellates, while diatom emergence did not respond to elevated temperature and light levels. In summer, total phytoplankton organic carbon emergence was orders of magnitude lower compared to spring and did not vary across treatments (GLM, Dev = 14.75, p > 0.12) averaging 3.0 μg m^−2^ (Fig. S2). The average biomass hatching of *Acartia* and *Temora* nauplii across all treatments was similar in spring (7.63 mg m^−2^) and summer (8.38 mg m^−2^) without any treatment effects (p > 0.81) (Fig. S2).

## DISCUSSION

To predict plankton bloom dynamics under climate change, an understanding of processes affecting life stage transitions, including recruitment of plankton from the sediment, is important. Our results show a substantial recruitment from benthic resting stages of cyanobacteria, diatoms, dinoflagellates, and copepods from coastal oxygenated sediments to the pelagic community that differs between seasons and the abiotic treatment. Phytoplankton germination is orders of magnitudes higher during the spring compared to the summer period, while copepod hatchlings varied between genera over the season. Activation of plankton resting stages are differentially affected by increasing temperature and light levels, indicating that climate-related physical changes can affect plankton dynamics through processes related to resting stage termination with potential consequences for bloom timing, taxa composition and trophic mismatch.

Germination of cyanobacteria increased about 10 times with a temperature increase from 3 to 5 °C but remained low under ambient (control) conditions at 3°C in spring. Increased light generated the same response, although the effect on germination were about half the value of those induced by temperature increase. Increased germination of *Aphanizomenon* cells at elevated temperature and light levels showed a more than 8-fold increase of inoculum from the sediment. This suggests that light and temperature conditions during early spring have strong effects on *Aphanizomenon* cell germination and consequently abundances in the water column as overwintering vegative cells are not consistently present in the water column (Fig. S3). For *Dolichospermum*, the contribution of cell germination from the sediment is the dominating driver to population growth as vegetative cells are absent in the water column over the winter (Fig. S3), which is in accordance with true dormancy in this genus (Rengefors *et al*., 2004). A 2°C temperature increase in early spring resulted in an almost 10-fold higher germination of dormant cells in *Dolichospermum*. Increasing germination from resting cells, when the water column is mixed in spring, enhances inoculum of cyanobacteria to the euphotic zone and likely benefits growth early in the year when nutrients are replete (Stahl-Delbanco, 2003; Suikkanen *et al*., 2010). This increases the competitive advantage of these cyanobacteria genera, overall contributing to stronger bloom formation during the summer period (Visser *et al*., 2016; Cottingham *et al*., 2021). Increasing global trends of cyanobacteria are related to their competitive growth over eukaryotic phytoplankton at higher water temperature (O’Neil *et al*., 2012). Our results demonstrate that higher germination from resting stages driven by bottom temperature increase and light availability in spring is an additional mechanism contributing to early inoculum of cyanobacteria from benthic resting stages and their competitive advantage with temperature warming.

Similarly, dinoflagellates emergence from the sediment was more than 2-fold at increasing temperature and the combined effect of light and temperature in spring compared to the control and light treatment. This contributes to higher cell proportions originating from the sediment, however, pelagic cells in the water column over winter and in March are manifold higher (about 13.0 cell L^−1^ over a 20 m water column emergence from the sediment vs up to 15 × 10^3^ cell L^−1^ in the water column, Fig. S3). This suggests that increased hatching with climate change enhances cell emergence from the sediment, but the impact is not as strong as for cyanobacteria because many of the dinoflagellate taxa overwinter in vegetive cells and spring blooms are most likely strongly affected by processes both in the upper water column and sediment. This is supported by observations showing the tight coupling of dinoflagellate shifts with water-column stratification (Hjerne *et al*., 2019). In contrast, cyanobacteria blooms are unrelated to abiotic processes in the water column (Kahru *et al*., 2018), suggesting the dominance of biological processes driving cyanobacteria bloom dynamics.

Surprisingly, germination of the diatom *Chaetoceros* was only high under control conditions, i.e., no light at ambient temperature, indicating that emergence of this spring bloom forming genus was already ongoing in early March. We found few cells of *Skeletonema* and *Thalassiosira*, other diatom genera forming the spring blooms (Höglander *et al*., 2004), despite them forming dormant cells (McQuoid and Godhe, 2004). *Skeletonema* dominate early spring blooms (Eilertsen *et al*., 1995) and recruitment from dormant cells may have already commenced at the time of the experiment. The overall abundance of diatom cell inoculum from the sediment was low in comparison to cell abundances in the water column (Fig. S3), also for the control treatment that reached the highest emergence numbers (about 2.8 cell L^−1^ over a 20 m water column). Our results suggest that germination of diatom cells from the sediment likely commenced before the sampling in March and that the seed bank was depleted by mid-March as diatom germination decreased substantially at day 14 of the experimental duration under cold and dark conditions (control). Interestingly, germination remained low throughout the experimental period at elevated temperature with a slight increase at day 14. This increase may be related to light stimuli during the experimental handling. This suggests that diatoms have narrow temperature optima under which emergence occurs, and favor regeneration at temperatures below 5°C. Decreased diatom emergence at elevated temperature and light levels is in line with their optimal growth under low-light and deep mixing conditions (Litchman and Klausmeier, 2008). These results suggest that temperature warming may decreases diatom cell germination from resting stages, lowering their overall competitive advantage with climate change. Moreover, the scarce impact of changing external conditions on diatom recruitment from dormant cells and low cell counts obtained in summer support the possibility of an internal clock or photoperiodic control on growth of diatom resting spores (Eilertsen *et al*., 1995).

Similar to phytoplankton, we observed few taxa of zooplankton recruitment from sediments, mainly consisting of the calanoid copepods *Acartia*, *Eurytemora* and to a lesser extent *Temora*. These findings are in agreement with the most common eggs identified in coastal sediments in the Baltic Sea (Viitasalo *et al*., 1994; Broman *et al*., 2019). However, the number of recruitments was not affected by elevated temperature and light levels, which likely relates to the traits of the resting eggs. *Acartia* (*A. bifilosa*) produce quiescent resting eggs that have no obligatory diapausing phase and emerge throughout the year (Katajisto, 2003). *Eurytemora* produce diapause eggs in autumn that overwinter in the sediment, while few individuals may also overwinter as adults in the water column (Katajisto *et al*., 1998). Our results support that recruitment is formed from both of these sources, namely by emergence of resting eggs during spring and early summer as observed with the hatching experiment, and reproduction of overwintering individuals as indicated by the presence of nauplii and adult *Eurytemora* in the water column during winter and spring (Fig. S4). For *Temora*, the development of the first generation is initiated by egg production of overwintering females, and our result suggests that recruitment from benthic resting stage of this copepod further contribute to population growth. Surprisingly, we observed low numbers of rotifer and cladoceran hatchlings in both periods, which is in contrast to the general assumption that populations of these taxa are recruited from hatchlings of resting stages (Kankaala, 1983). Overall, results from copepod emergence suggest that shifts in bloom dynamics are mainly related to processes in the water column that affect pelagic life stages rather than quantitative changes in benthic recruitment due to changing light or temperature conditions.

We find that temperature had the largest effect on cyanobacteria and dinoflagellate cell germination. The temperature effect was significantly higher compared to the light treatment and the combined effect of light and temperature in cyanobacteria, and for some taxa the combined factors resulted in counteracting effects with lower germination or emergence than single factor effects. This is contrary to our expectation of additive effects of temperature and light, suggesting that these two drivers have differential or even counteracting effects on plankton recruitment from resting stages. Overall, our results suggest that climate warming favors germination and/or growth of benthic dinoflagellates and cyanobacteria life stages. In contrast, diatom emergence from resting stages may be reduced at elevated bottom temperature, while copepod emergence is less affected by change in temperature and light levels. Our experimental results are in line with observed phenological findings of earlier and stronger dinoflagellate and cyanobacteria blooms, declines in spring diatoms, while copepod phenology remained unchanged in the central Baltic sea over the last decades (Kahru and Elmgren, 2014; Hjerne *et al*., 2019; Jan, K *et al*., in prep). At the same time, bottom temperature at 20 m has increased for about 1.7 °C over the last 20 years (Fig. S5) and sea ice extent has decreased (Hjerne *et al*., 2019). Our results adds mechanistic understanding to bloom dynamics and suggests that higher germination of cyanobacteria in spring increases the standing stock during summer when cyanobacteria typically have a competitive advantage over other phytoplankton taxa at higher temperature and nitrogen limitation (Litchman and Klausmeier, 2008; Paerl and Huisman, 2008). Similarly, earlier germination of dinoflagellate resting stages with climate warming contributes to higher abundances during their bloom periods, increasing competition with diatoms that typically dominate initially during the bloom (Angeler *et al*., 2015).

Our results further suggests that climate change can affect the timing and magnitude of organic carbon flux to the water column. Temperature warming and higher light levels advances the flux of phytoplankton carbon in spring. Here, we estimate that the flux of organic carbon from sediment to the water column is roughly 72.6 μg C m^−2^ week^−1^ for phytoplankton and 4.5 mg m^−2^ week^−1^ for copepod nauplii in spring. By comparison, the remineralized dissolved inorganic carbon efflux from aerobic and anaerobic respiration in these sediments during June is between 10 and 17 mg m^−2^ week^−1^ (Bonaglia *et al*., 2013; Fredriksson *et al*., in review), and therefore of the same magnitude as the observed emergence of resting stages. Sedimentation rates are orders of magnitude higher, with spring bloom sedimentation estimated to about 500 mg C m^−2^ week^−1^ (Höglander *et al*., 2004) and summer sedimentation rates about 14 to 21 mg C m^−2^ week^−1^ (Heiskanen and Kononen, 1994). This suggests that organic flux from sediment to the open water is minor compared to inputs of sedimenting material to the benthos. However, high spatial and temporal variability can be expected, given the diverse habitat structures of coastal systems. Moreover, bottom water temperatures increase with climate warming will not only affect inorganic carbon flux (Jansen *et al*., 2022) but also fluxes resulting of biological-mediated processes from the sediment to the water column.

Hatching and germination experiments under climate change scenarios provide valuable insight into controlling mechanisms of recruitment, but the experimental set-ups also had limitations. Our experiment did not allow the separation of phyto- and zooplankton organisms, and low numbers of phytoplankton cells observed during the summer may be explained by grazing impacts. However, we assume low grazing pressure from nauplii as they have longer development times at low temperatures and reduced energy requirements (Leandro, Queiroga, *et al*., 2006; Leandro, Tselius, *et al*., 2006). In summer, reduced cell abundances at elevated temperatures may be due to the presence of adult *Eurytemora* individuals that were not completely removed by siphoning off the initial water, most likely due to the agile swimming capability of this diel vertically migrating copepod (Holliland *et al*., 2012). For the same reason, we were not able to estimate *Eurytemora* nauplii from resting stages in summer. However, our results presented here are a major step towards a better mechanistic understanding of climate impacts on plankton bloom dynamics.

## CONCLUSIONS

Our study provides evidence that changing temperature and light affects benthic resting stage release with consequences for phytoplankton bloom timing, their magnitude and taxa composition. In particular, our study provides a mechanistic explanation that relates emergence and resting stage traits to observed increases of dinoflagellate and cyanobacteria blooms in the water column, and declining trend of diatoms with temperature warming. In contrast, emergence of copepod nauplii from the sediment remained unaffected by changing temperature and light levels, indicating that zooplankton bloom dynamics are more related to the type of resting stage and processes occurring in the water column. Moreover, our results show that the competitive advantage of cyanobacteria and dinoflagellates under climate warming is related to earlier germination of resting stages. However, copepod emergence is unaffected by change in temperature, increasing the potential of trophic mismatch with the spring bloom dynamics.

## Supplementary Material

Hedberg_et_al_Suppl_Information-Final_fbad060

## Data Availability

Data are available in the Dryad repository: https://doi.org/10.5061/dryad.8cz8w9gwj.
