# Supplementary material for: Climate change effects on plankton recruitment from coastal sediments
Source: J Plankton Res. 2024 Feb 15;46(2):117–25. doi: 10.1093/plankt/fbad060 (PMC10987100; doi:10.1093/plankt/fbad060)

SUPPLEMENTARY INFORMATION

# Climate change effects on plankton recruitment from coastal sediments

P. Hedberg[1], M. Olsson[1], H. Höglander[1], V. Brüchert[2,3], M. Winder[1,3]

[1]Department of Ecology, Environment and Plant Sciences, Stockholm University, Stockholm, Sweden

[2]Department of Geological Sciences, Stockholm University, Sweden

[3]Bolin Centre for Climate Research, Stockholm University, Sweden

**Fig. S1**. Counts of adult copepod individuals from Baltic Sea coastal sediments in week 1 (day 7) and week 2 (day 14) of the experiment during the spring (March) and summer (June) period. Counts are shown for the following treatments: control (CTRL) kept dark at *in situ* temperature (3 °C in spring, 9 °C in summer), an increase of +2 °C (T), weak green light (L), and a combination of elevated temperature and light (T+L). Filled symbols are total counts, open symbols are the dominating species, contributing to more than 95% of total counts.

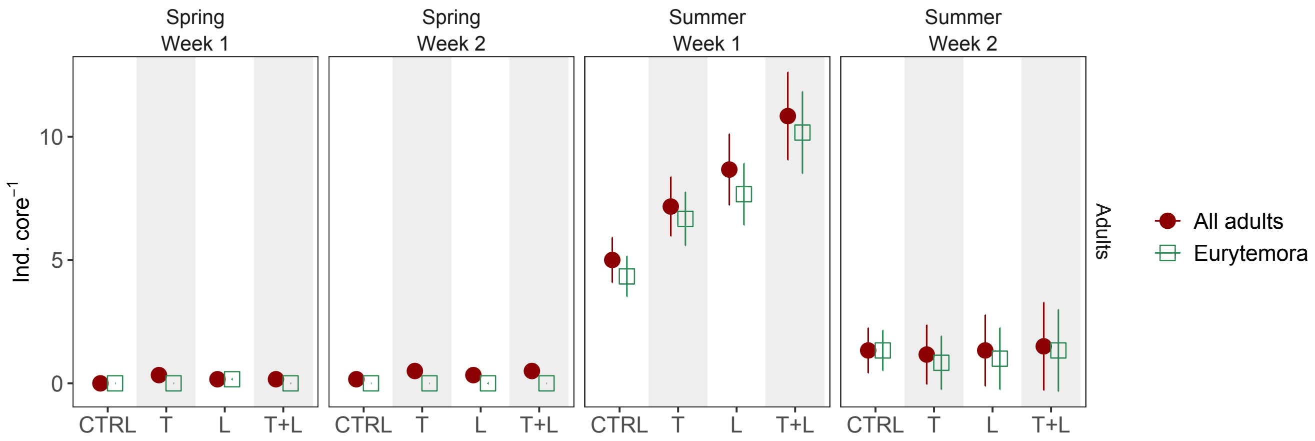

**Fig. S2.** Mean (± S.E.) biomass from recruitment of phytoplankton (in C m$^{-2}$; top) and copepod nauplii (in wet weight; bottom) from Baltic Sea coastal sediments during the spring (left column) and summer (right column) period. Phytoplankton include cyanobacteria, diatoms and dinoflagellates. See Fig. S1 for treatment description. Filled symbols are total biomass estimates for phytoplankton. Note, *Eurytemora* carbon estimation are excluded from week 1 in summer.

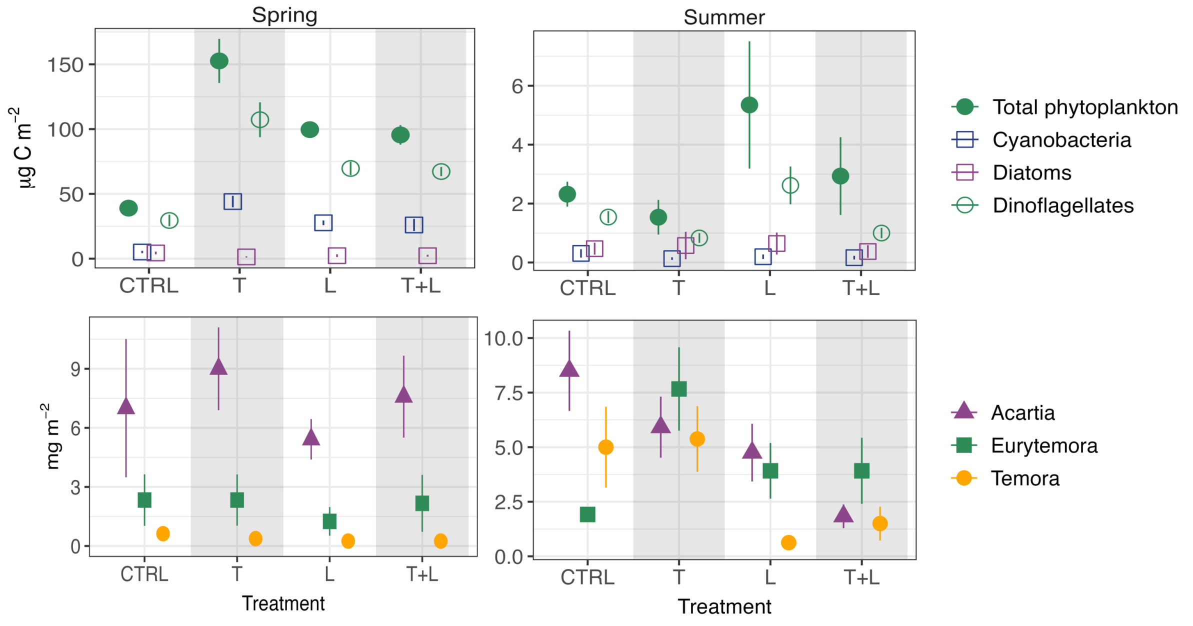

**Fig. S3.** Phytoplankton counts during the winter month of January, February and March for the dominant species identified in the experiment. Phytoplankton data are from the nearest monitoring station B1 with a depth of 40 m. Values are shown from 20 m depth for the period 2009 to 2021 available at https://sharkweb.smhi.se/. The temporal resolution of the underlying sampling is monthly during winter (Jan-Feb) and weekly during the spring bloom (Mar). Note that zero values are not shown.

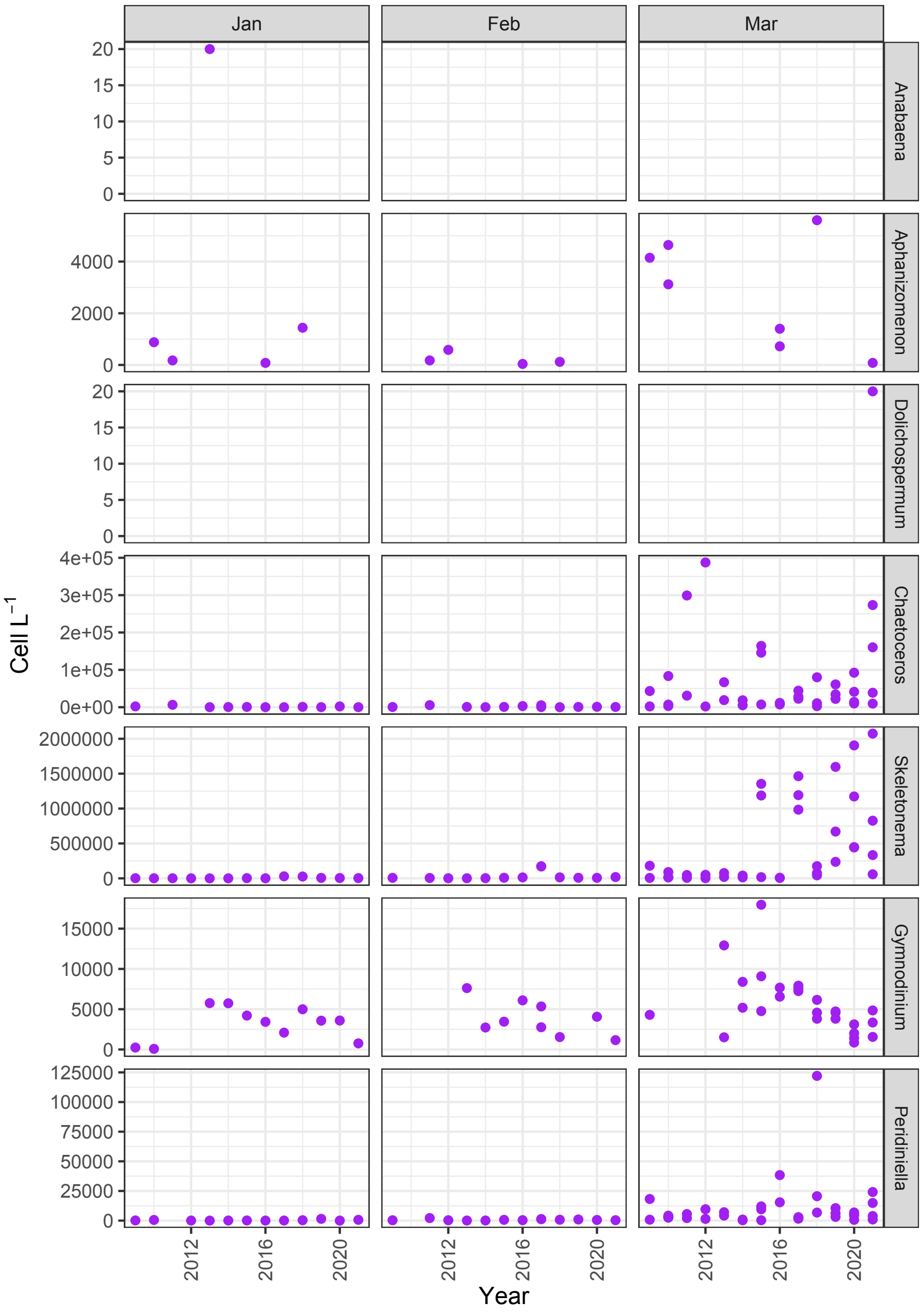

**Fig. S4.** Copepod counts from January to June for the dominant species identified in the experiment for nauplii and adult stages. Zooplankton data are from the nearest monitoring station B1 with a depth of 40 m. Values are shown over the 0 to 30 m depth strata for the period 2008 to 2021 available at https://sharkweb.smhi.se/. The temporal resolution of the underlying sampling is monthly during winter (Nov-Feb), weekly during the spring bloom (Mar-Apr) and bi-weekly during the remaining season. Note that zero value are not shown.

Nauplii

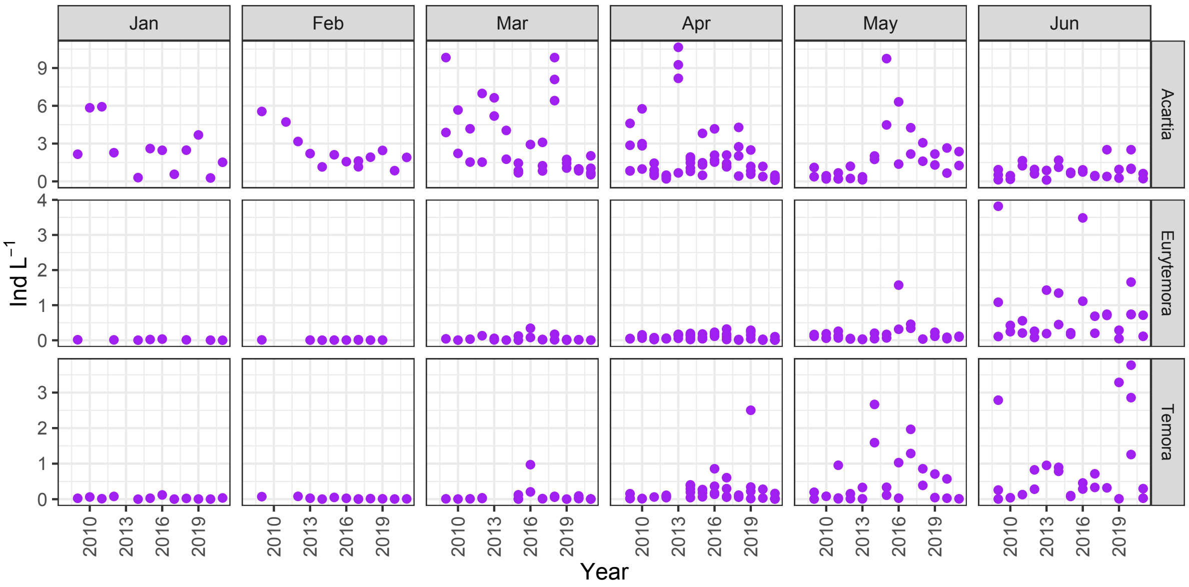


Adult

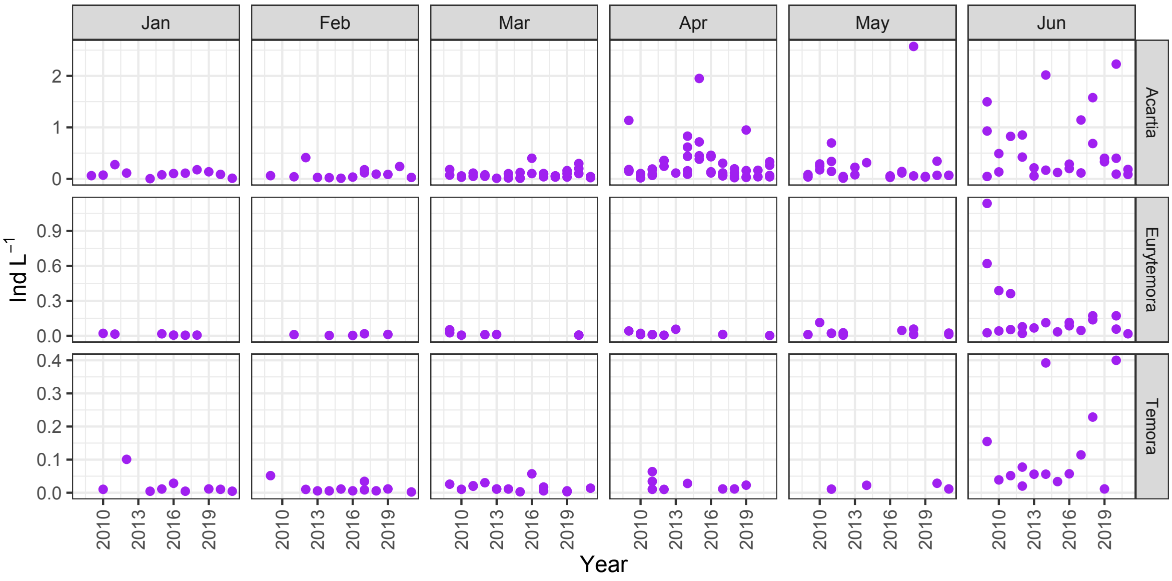

**Fig. S5.** Monthly mean temperature (left) and temperature anomalies (right) at 20 m depth from the nearest monitoring station B1. Temperature increased significantly in March (linear regression, slope = 0.07, $p = 0.018$), April (slope = 0.06, $p = 0.033$), May (slope = 0.06, $p = 0.048$), and June (slope = 0.12, $p = 0.022$). Temperature data include the period 1998 to 2021 available at (https://sharkweb.smhi.se/). The temporal resolution of the underlying sampling is weekly during the spring bloom (Mar-Apr) and bi-weekly during the remaining season.

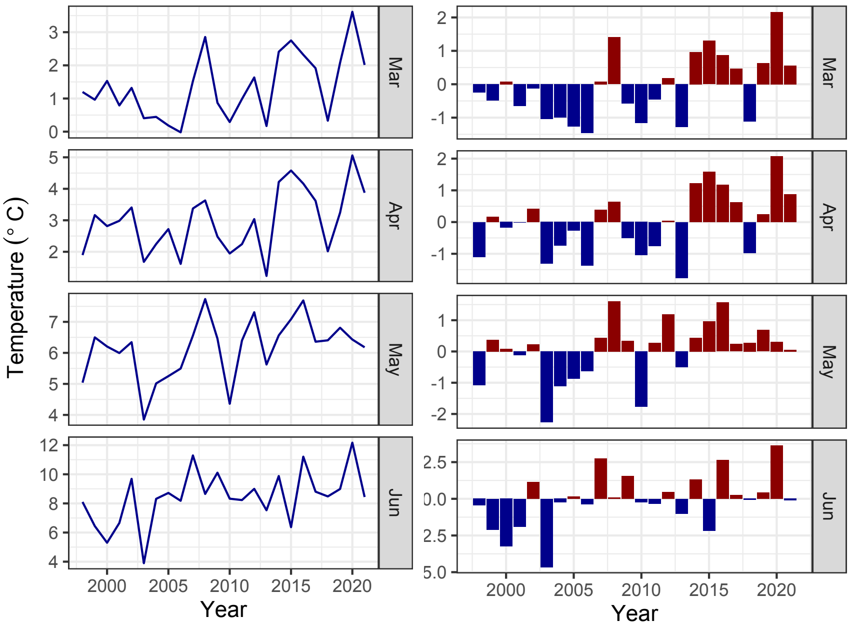

Supplement: Hedberg_et_al_Suppl_Information-Final_fbad060 [file hedberg_et_al_suppl_information-final_fbad060.pdf]
